# Robust Covariance Estimators Based on Information Divergences and Riemannian Manifold

**DOI:** 10.3390/e20040219

**Published:** 2018-03-23

**Authors:** Xiaoqiang Hua, Yongqiang Cheng, Hongqiang Wang, Yuliang Qin

**Affiliations:** School of Electronic Science, National University of Defence Technology, Changsha 410073, China

**Keywords:** information divergence, Riemannian manifold, covariance estimation, mean estimator, heterogeneous clutter

## Abstract

This paper proposes a class of covariance estimators based on information divergences in heterogeneous environments. In particular, the problem of covariance estimation is reformulated on the Riemannian manifold of Hermitian positive-definite (HPD) matrices. The means associated with information divergences are derived and used as the estimators. Without resorting to the complete knowledge of the probability distribution of the sample data, the geometry of the Riemannian manifold of HPD matrices is considered in mean estimators. Moreover, the robustness of mean estimators is analyzed using the influence function. Simulation results indicate the robustness and superiority of an adaptive normalized matched filter with our proposed estimators compared with the existing alternatives.

## 1. Introduction

Covariance estimation plays an important role in adaptive signal processing, such as multichannel signal processing [1,2], space-time adaptive processing (STAP) [3,4], and radar target detection. Conventional covariance estimation methods, derived from the maximum-likelihood (ML) of the clutter data, are based on the assumption that the clutter remains stationary and homogeneous during the adaptation process. However, in real heterogeneous clutter, the number of the sample data where the clutter is homogeneous is very limited, and the estimation performance is seriously degraded. Therefore, it is necessary and important to improve the performance of estimated covariance in heterogeneous clutter.

A commonly used strategy to ameliorate the performance of estimated covariance is to exploit some a priori information about the clutter environment. For instance, the geographic information is used for covariance estimation, and the performance of target detection with the estimator is significant improved [5]. In [6], the authors employ the Bayesian method to perform the target detection in interference environment, where the unknown covariance matrix is assumed to follow a suitable probability model. In [7,8], a Bayesian framework is used together with the structural information about the estimated covariance. In [9], a condition number upper-bound constraint is imposed on the problem of covariance estimation to achieve a signal-to-noise ratio improvement. Moreover, a symmetrically structured spectral density is constrained on the covariance estimation, and these results show the superiority of the estimator [10]. These mentioned methods rely on the knowledge of statistics characterization of the clutter data. However, the probability distribution of the whole environment is difficult to obtain, and a mismatched distribution results in a remarkable degradation of the estimation performance in heterogeneous clutter.

Many covariance estimation algorithms derived from the geometry of matrix space, not resorting to the statistical characterization of the sample data, are reported in the literature. For instance, the Riemannian mean is used for monitoring the wake turbulence [11,12] and target detection in HF and X-band radar [13]. In [14,15,16], Riemannian mean and median are employed for covariance estimation in STAP, and results have shown that the projection algorithm with Riemannian mean can yield significant performance gains. In [17,18], some geometric barycenter and medians are proposed for radar training data selection in homogeneous environment. In recent times, we have explored information divergence means and medians for target detection in non-Gaussian clutter [19,20,21]. Moreover, in image processing applications, Bhattacharyya mean and median are used for filtering and clustering of diffusion tensor magnetic resonance image [22,23]. In [24], the Log-Euclidean mean, together with the reproducing kernel Hilbert mapping, is used for texture recognition. These geometric approaches have achieved good performances.

In this paper, a class of covariance estimators based on information divergences is proposed in heterogeneous clutter. In particular, six means related to geometric measures are derived on the Riemannian manifold of Hermitian positive-definite (HPD) matrices. These means do not rely on the knowledge of statistics characterization of sample data, and the geometry of the matrix space is considered. Moreover, the robustness of means is analyzed with injected outliers via the influence function. Simulation results are given to validate the superiority of proposed estimators.

The rest of this paper is organized as follows. In Section 2, we reformulate the problem of covariance estimation on the Riemannian manifold. In Section 3, the geometry of Riemannian manifold of HPD matrices is presented, in particular, six distance measures are given on the Riemannian manifold, and means associated with these measures are derived. The robustness of means is analyzed via the influence function in Section 4. Then, we evaluate performances of an adaptive normalized matched filter with geometric means as well as the normalized sample covariance matrix in Section 5. Finally, conclusion is provided in Section 6.

### Notation

Here is some notation for the descriptions of this article. A matrix A and a vector x are noted as uppercase bold and lowercase bold, respectively. The conjugate transpose of matrix A is denoted as $A^{H}$. tr(A) is the trace of matrix A. |A| is the determinant of matrix A. I denotes the identity matrix. Finally, E(.) denotes the statistical expectation.

## 2. Problem Reformulated on the Riemannian Manifold

A heterogeneous environment is considered for covariance estimation. For a set of *K* secondary data $\left\{r_{1},\ldots ,r_{K}\right\}$, the normalized sample covariance matrix (NSCM) $\hat{R}$ based on the ML of probability distribution of the sample data is estimated as,

(1) $$\begin{array}{c} \hat{R}=\frac{1}{K}\sum_{k=1}^{K}\frac{r_{k}r_{k}^{H}}{\frac{1}{N}\;r_{k}^{H}r_{k}}=\frac{1}{K}\sum_{k=1}^{K}R_{k},\\ R_{k}=\frac{r_{k}r_{k}^{H}}{\frac{1}{N}\;r_{k}^{H}r_{k}} \end{array}$$

where *N* is the dimension of the vector $r_{k}$. $r_{k},k=1,\ldots ,K$ are modeled as a compound-Gaussian random vector, and can be expressed as,

(2) $$\begin{array}{c} r_{k}=\sqrt{\tau_{k}}g_{k},k=1,\ldots ,K \end{array}$$

where $\tau_{k}$ is a nonnegative scalar random variable, and $g_{k}$ is a N-dimensional circularly symmetric zero-mean vectors with an arbitrary joint statistical distribution and sharing the same covariance matrix,

(3) $$\begin{array}{c} E[g_{k}g_{k}^{H}]=\Sigma ,k=1,\ldots ,K \end{array}$$

It is clear from Equation (1) that the NSCM is the arithmetic mean of *K* auto-covariance matrices $R_{k}$ of rank one. Since the knowledge of probability distribution of the whole environment is difficult to obtain in heterogeneous clutter, the performance of NSCM is severely degraded. Actually, these *K* auto-covariance matrices lie in a non-linear Hermitian matrix space, as the sample data is complex. It is well known that HPD matrices form a differentiable Riemannian manifold [25], that is the most studied example of a manifold with non-positive curvature [26]. In order to facilitate the analysis, the matrix $R_{k}$ is transformed to the positive- definite matrix $P_{k}$ using the following three ways:

(1) $P_{k}$ is obtained by adding an identity matrix I, as $P_{k}=R_{k}+I$;
(2) A Toeplitz HPD matrix $T_{k}$ is utilized. As in [13], $T_{k}$ can be expressed as,
  (4) $$\begin{array}{c} T_{k}=\left[r_{k}r_{k}^{H}\right]=\left[\begin{array}{c} \begin{array}{cccc} c_{0} & {\bar{c}}_{1} & \cdots & {\bar{c}}_{N-1} \end{array}\\ \begin{array}{cccc} c_{1} & c_{0} & \cdots & {\bar{c}}_{N-2} \end{array}\\ \begin{array}{cccc} \vdots & \ddots & \ddots & \vdots \end{array}\\ \begin{array}{cccc} c_{N-1} & \cdots & c_{1} & c_{0} \end{array} \end{array}\right],c_{k}=\left[r_{i}{\bar{r}}_{i+k}\right],0\leq k\leq N-1,0\leq i\leq N-1 \end{array}$$
  where $c_{k}$ denotes the correlation coefficient of sample data, and ${\bar{c}}_{i}$ is the complex conjugate of $c_{i}$. $c_{k}$ can be computed as,
  (5) $$\begin{array}{c} c_{k}=\frac{1}{N-k}\sum_{j=1}^{N-1-k}r_{j}{\bar{r}}_{j+k},0\leq k\leq N-1 \end{array}$$
(3) The HPD matrix $S_{k}$ is the solution of the optimization problem as follows [17],
  (6) $$\begin{array}{c} \left\{\begin{array}{c} \min_{S_{k}}\;{∥r_{k}r_{k}^{H}/r_{k}^{H}r_{k}\;-S_{k}∥}^{2}\\ S_{k}\geq I\\ \frac{\lambda_{\max}\left(S_{k}\right)}{\lambda_{\min}\left(S_{k}\right)}\leq \kappa_{M} \end{array}\right. \end{array}$$

The optimal solution can be given by [17],

(7) $$\begin{array}{c} S_{k}=U_{k}\Lambda_{k}U_{k}^{H}\\ \Lambda_{k}=diag\left(\left[\kappa_{M}\lambda_{k},\lambda_{k},\ldots ,\lambda_{k}\right]\right)\\ \lambda_{k}=\max\left(1,\frac{{∥r_{k}∥}^{2}\kappa_{M}}{\kappa_{M}^{2}+N-1}\right) \end{array}$$

where $U_{k}$ is a unitary matrix of the eigenvectors of $r_{k}r_{k}^{H}$ with the first eigenvector corresponding to the eigenvalue $∥r_{k}{∥}^{2}$. The $\kappa_{M}$ is the condition number.

According to above transformations, we can obtain the HPD matrix. Then, the problem of covariance estimation can be reformulated on the Riemannian manifold. In general, for a set of *m* scalar numbers $x_{1},\ldots ,x_{m}$, the arithmetic mean is defined as the minimum of sum of squared distances to the given point *x*,

(8) $$\begin{array}{c} \bar{x}=\frac{1}{m}\sum_{i=1}^{m}x_{i}=\underset{x>0}{\arg\min}\;\sum_{i=1}^{m}{\left|x-x_{i}\right|}^{2} \end{array}$$

**Figure 1:**
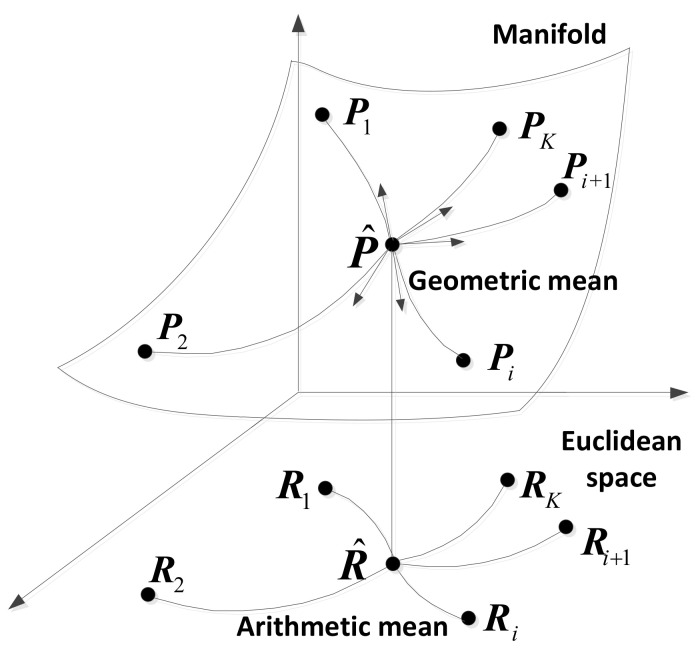
The geometric mean and the arithmetic mean.

From Equation (8), we can understand the arithmetic mean from a geometric viewpoint. Similar to Equation (8), for *K* HPD matrices $P_{1},\ldots ,P_{K}$, the mean related to a measure can be defined as,

(9) $$\begin{array}{c} \hat{P}=\underset{P}{\arg\min}\frac{1}{K}\sum_{k=1}^{K}d^{2}\left(P,P_{k}\right) \end{array}$$

where d(.,.) denotes the measure. It is worth pointing out that the arithmetic mean Equation (1) is obtained, when *d* is the Frobenius norm and $P_{k}$ is replaced by $R_{k}$. The difference between the arithmetic mean and the geometric mean is shown in Figure 1. As illustrated in Figure 1, the geometric median is performed on the Riemannian manifold of HPD matrices with a non-Euclidean metric, whereas the arithmetic mean is considered in the Euclidean space. The difference implies that the different geometric structures are considered in these two estimators.

## 3. The Geometry of Riemannian Manifold of HPD Matrices

In this Section, the fundamental mathematic knowledge related to this paper is presented. Firstly, the Riemannian manifold of HPD matrices is introduced. Then, six distance measures are presented. Finally, the means related to measures are derived.

### 3.1. The Riemannian Manifold of HPD Matrices

Let $H\left(n\right)=\left\{A|A=A^{H}\right\}$ denotes the space of n×n Hermitian matrix. For a Hermitian matrix A, if the quadratic form $x^{H}Ax>0,\forall x\in C\left(n\right)$, then, A is an HPD matrix, where C(n) is the space of *n*-dimensional complex vector. All n×n HPD matrices consist of a positive-definite Hermitian matrix space P(n),

(10) $$\begin{array}{c} P(n)=\{A|A>0,A\in H(n)\} \end{array}$$

forms a Riemannian manifold of dimension n(n+1)/2 with a constant non-positive curvature [26]. For a point A on the Riemannian manifold, the infinitesimal arclength between A and A+dA is given by [27],

(11) $$\begin{array}{c} ds:={\left(tr{\left(A^{-1}dA\right)}^{2}\right)}^{1/2}={∥A^{-1/2}dAA^{-1/2}∥}_{F} \end{array}$$

where ds defines a metric on the Riemannian manifold [27]. ${∥.∥}_{F}$ is the Frobenius norm of a matrix. The inner product and corresponding norm on the tangent space at the point A can be defined as [28],

(12) $$\begin{array}{c} {\langle P_{1},P_{2}\rangle }_{A}=tr\left(A^{-1}P_{1}A^{-1}P_{2}\right),{∥P_{1}∥}_{A}={\langle P_{1},P_{2}\rangle }_{A}^{1/2} \end{array}$$

For two points $P_{1}$ and $P_{2}$ on the Riemannian manifold, the affine invariant (Riemannian) distance is given by [29],

(13) $$\begin{array}{c} d_{R}^{2}\left(P_{1},P_{2}\right)={∥logm\left(P_{1}^{-1/2}P_{2}P_{1}^{-1/2}\right)∥}_{F}^{2} \end{array}$$

where logm is the logarithmic map on the Riemannian manifold of HPD matrices.

### 3.2. The Geometric Measure on the Riemannian Manifold

In addition to the Riemannian distance, a lot of distance or information divergences can be used as the measurement on the Riemannian manifold. Here, five geometric measures are presented in the following.

(1) Log-Euclidean distance 

The Log-Euclidean distance is also a geodesic distance. It is defined on the tangent space at a point on the Riemannian manifold, which is isomorphic and diffeomorphic to the tangent space identified with a Hermitian matrix space. For two points $P_{1}$ and $P_{2}$, the Log-Euclidean distance $d_{LE}\left(P_{1},P_{2}\right)$ is given by [30],

(14) $$\begin{array}{c} d_{LE}\left(P_{1},P_{2}\right)={∥logm\left(P_{1}\right)-logm\left(P_{2}\right)∥}_{F} \end{array}$$

(2) Hellinger distance

The Hellinger distance is a special case of the α-divergence with α=0. Given two points $P_{1}$ and $P_{2}$, the Hellinger distance $d_{H}\left(P_{1},P_{2}\right)$ is [31],

(15) $$\begin{array}{c} d_{H}\left(P_{1},P_{2}\right)=\sqrt{2-2\frac{{\left|P_{1}\right|}^{1/4\;}{\left|P_{2}\right|}^{1/4\;}}{{\left|\frac{1}{2}\left(P_{1}+P_{2}\right)\right|}^{1/2\;}}} \end{array}$$

(3) Kullback-Leibler divergence

It is well known that the Kullback-Leibler (KL) divergence is the most widely used measure on the Riemannian manifold. The KL divergence is also a special case of the α-divergence with α=±1. In addition, the KL divergence is called the Stein loss or the log-determinant divergence. The KL divergence $d_{KL}\left(P_{1},P_{2}\right)$ between two points $P_{1}$ and $P_{2}$ can be given by [32],

(16) $$\begin{array}{c} d_{KL}\left(P_{1},P_{2}\right)=tr\left(P_{2}^{-1}P_{1}-I\right)-\log\left|P_{2}^{-1}P_{1}\right| \end{array}$$

(4) Bhattacharyya distance 

The Bhattacharyya distance is a common used measure, and has been used in medical image segmentation [22]. In particular, the Bhattacharyya distance is a Jensen version of the KL divergence. For two points $P_{1}$ and $P_{2}$, the Bhattacharyya distance $d_{B}\left(P_{1},P_{2}\right)$ can be given by,

(17) $$\begin{array}{c} d_{B}\left(P_{1},P_{2}\right)=2\sqrt{\log\frac{\left|\frac{P_{1}+P_{2}}{2}\right|}{\sqrt{\left|P_{1}\right|\left|P_{2}\right|}}} \end{array}$$

(5) Symmetrized Kullback-Leibler divergence

The symmetrized Kullback-Leibler (SKL) divergence is a Jeffreys divergence [33]. It behaves as the square of a distance; however, it is not a distance, as the triangle inequality does not hold. Given two points $P_{1}$ and $P_{2}$, the SKL $d_{SKL}\left(P_{1},P_{2}\right)$ between them is,

(18) $$\begin{array}{c} d_{SKL}\left(P_{1},P_{2}\right)=\frac{1}{2}\left\{d_{KL}\left(P_{1},P_{2}\right)+d_{KL}\left(P_{2},P_{1}\right)\right\}=\frac{1}{2}tr\left(P_{2}^{-1}P_{1}+P_{1}^{-1}P_{2}-2I\right) \end{array}$$

### 3.3. The Geometric Mean for A Set of HPD Matrices

In this Section, the means related to the above mentioned six measures are derived using the fix-point algorithm. This work has been done in our previous article [19]. In the following, six means are presented in Table 1.

## 4. Robustness Analysis of Geometric Means

This section is devoted to analyzing the robustness of geometric means via the influence function. Let $\bar{P}$ be the mean, associated with a measure, of *m* HPD matrices $\left\{P_{1},\ldots ,P_{m}\right\}$. $\tilde{P}$ is the mean by adding a set of *n* outliers $\left\{Q\ldots \ldots ,Q_{n}\right\}$ with a weight ε(ε≪1) to $\left\{\ldots_{1},\ldots ,P_{m}\right\}$. Then, we can define $\tilde{P}=\bar{P}+\varepsilon H\left(Q\right)$, H(Q) denotes the influence function. In the following, seven propositions are presented.

**Proposition** **1.** *The influence function of arithmetic mean related to the Frobenius norm, of m HPD matrices $\left\{P_{1},\ldots ,P_{m}\right\}$ and n outliers $\left\{Q\ldots \ldots ,Q_{n}\right\}$ is given by,*

(19) $$\begin{array}{c} H\left(Q\right)=\frac{1}{n}\sum_{i=1}^{n}Q_{i}-\bar{P} \end{array}$$

**Proof** **of** **Proposition** **1.** Let F(P) be the objection function,

(20) $$\begin{array}{c} F\left(P\right)=\left(1-\varepsilon \right)\frac{1}{m}\sum_{i=1}^{m}∥P-P_{i}{∥}_{F}^{2}+\varepsilon \frac{1}{n}\sum_{j=1}^{n}∥P-Q_{j}{∥}_{F}^{2} \end{array}$$

The derivative of objection function F(P) is,

(21) $$\begin{array}{c} \nabla F\left(P\right)=\left(1-\varepsilon \right)\frac{1}{m}\sum_{i=1}^{m}2\left(P-P_{i}\right)+\varepsilon \frac{1}{n}\sum_{j=1}^{n}2\left(P-Q_{j}\right) \end{array}$$

Note that $\tilde{P}$ is the mean of *m* matrices and *n* outliers, and $\bar{P}$ is the mean of *m* matrices, then, we have,

(22) $$\begin{array}{cc} & \tilde{P}=\underset{P}{\arg\min}\;F\left(P\right)\\ \Rightarrow & \nabla F\left(\tilde{P}\right)=\left(1-\varepsilon \right)\frac{1}{m}\sum_{i=1}^{m}2\left(\tilde{P}-P_{i}\right)+\varepsilon \frac{1}{n}\sum_{j=1}^{n}2\left(\tilde{P}-Q_{j}\right)=0 \end{array}$$

and

(23) $$\begin{array}{cc} & \bar{P}=\underset{P}{\arg\min}\;G\left(P\right),G\left(P\right)=\frac{1}{m}\sum_{i=1}^{m}{∥P-P_{i}∥}_{F}^{2}\\ \Rightarrow & \nabla F\left(\bar{P}\right)=\frac{1}{m}\sum_{i=1}^{m}2\left(\bar{P}-P_{i}\right)=0 \end{array}$$

Substitute $\tilde{P}=\bar{P}+\varepsilon H\left(Q\right)$ into Equation (22), and we have

(24) $$\begin{array}{cc} & 2\left(1-\varepsilon \right)\frac{1}{m}\sum_{i=1}^{m}\left(\bar{P}+\varepsilon H\left(Q\right)-P_{i}\right)+2\varepsilon \frac{1}{n}\sum_{j=1}^{n}\left(\bar{P}+\varepsilon H\left(Q\right)-Q_{j}\right)=0\\ \Rightarrow & \left(1-\varepsilon \right)\frac{1}{m}\sum_{i=1}^{m}\left(\bar{P}-P_{i}\right)+\left(1-\varepsilon \right)\varepsilon H\left(Q\right)+\varepsilon \frac{1}{n}\sum_{j=1}^{n}\left(\bar{P}+\varepsilon H\left(Q\right)-Q_{j}\right)=0\\ \Rightarrow & \varepsilon H\left(Q\right)-\varepsilon^{2}H\left(Q\right)+\varepsilon \frac{1}{n}\sum_{j=1}^{n}\left(\bar{P}-Q_{j}\right)+\varepsilon^{2}H\left(Q\right)=0\\ \Rightarrow & H\left(Q\right)=\frac{1}{n}\sum_{j=1}^{n}\left(Q_{j}-\bar{P}\right) \end{array}$$

☐

**Proposition** **2.** *The influence function of Riemannian mean related to the Riemannian distance, of m HPD matrices $\left\{P_{1},\ldots ,P_{m}\right\}$ and n outliers $\left\{Q\ldots \ldots ,Q_{n}\right\}$ is given by,*

(25) $$\begin{array}{c} H\left(Q\right)=-\frac{1}{n}\sum_{j=1}^{n}log\left(Q_{j}^{-1}\bar{P}\right)\bar{P} \end{array}$$

**Proof** **of** **Proposition** **2.** Let F(P) be the objection function,

(26) $$\begin{array}{c} F\left(P\right)=\left(1-\varepsilon \right)\frac{1}{m}\sum_{i=1}^{m}∥\log\left(P_{i}^{-1}P\right){∥}_{F}^{2}+\varepsilon \frac{1}{n}\sum_{j=1}^{n}∥\log\left(Q_{j}^{-1}P\right){∥}_{F}^{2} \end{array}$$

As $\tilde{P}$ is the mean of *m* matrices and *n* outliers, and $\bar{P}$ is the mean of *m* matrices, then, we have,

(27) $$\begin{array}{cc} & \tilde{P}=\underset{P}{\arg\min}\;F\left(P\right)\\ \Rightarrow & \nabla F\left(\tilde{P}\right)=2\left(1-\varepsilon \right)\frac{1}{m}\sum_{i=1}^{m}\log\left(P_{i}^{-1}\tilde{P}\right){\tilde{P}}^{-1}+2\varepsilon \frac{1}{n}\sum_{j=1}^{n}\log\left(Q_{j}^{-1}\tilde{P}\right){\tilde{P}}^{-1}=0\\ \Rightarrow & \left(1-\varepsilon \right)\frac{1}{m}\sum_{i=1}^{m}\log\left(P_{i}^{-1}\tilde{P}\right)+\varepsilon \frac{1}{n}\sum_{j=1}^{n}\log\left(Q_{j}^{-1}\tilde{P}\right)=0 \end{array}$$

and

(28) $$\begin{array}{cc} & \bar{P}=\underset{P}{\arg\min}\;G\left(P\right),G\left(P\right)=\frac{1}{m}\sum_{i=1}^{m}{∥\log\left(P_{i}^{-1}P\right)∥}_{F}^{2}\\ \Rightarrow & \nabla G\left(\bar{P}\right)=\frac{1}{m}\sum_{i=1}^{m}2\log\left(P_{i}^{-1}\bar{P}\right)){\bar{P}}^{-1}=0\Rightarrow \frac{1}{m}\sum_{i=1}^{m}\log\left(P_{i}^{-1}\bar{P}\right))=0 \end{array}$$

Using the Taylor expansion on $\tilde{P}=\bar{P}+\varepsilon H\left(Q\right)$, and we have

(29) $$\begin{array}{c} \log\left(P_{i}^{-1}\tilde{P}\right)=\log\left(P_{i}^{-1}\bar{P}\right)+\varepsilon H\left(Q\right)P_{i}^{-1}{\left(P_{i}^{-1}\bar{P}\right)}^{-1}=\log\left(P_{i}^{-1}\bar{P}\right)+\varepsilon H\left(Q\right){\bar{P}}^{-1}\\ \log\left(Q_{j}^{-1}\tilde{P}\right)=\log\left(Q_{j}^{-1}\bar{P}\right)+\varepsilon H\left(Q\right)Q_{j}^{-1}{\left(Q_{j}^{-1}\bar{P}\right)}^{-1}=\log\left(Q_{j}^{-1}\bar{P}\right)+\varepsilon H\left(Q\right){\bar{P}}^{-1} \end{array}$$

Substitute Equations (28) and (29) into Equation (27), and we have

(30) $$\begin{array}{cc} & \left(1-\varepsilon \right)\frac{1}{m}\sum_{i=1}^{m}\left(\log\left(P_{i}^{-1}\bar{P}\right)+\varepsilon H\left(Q\right){\bar{P}}^{-1}\right)+\varepsilon \frac{1}{n}\sum_{j=1}^{n}\left(\log\left(Q_{j}^{-1}\bar{P}\right)+\varepsilon H\left(Q\right){\bar{P}}^{-1}\right)=0\\ \Rightarrow & \left(1-\varepsilon \right)\frac{1}{m}\sum_{i=1}^{m}\log\left(P_{i}^{-1}\bar{P}\right)+\left(1-\varepsilon \right)\varepsilon H\left(Q\right){\bar{P}}^{-1}+\varepsilon \frac{1}{n}\sum_{j=1}^{n}\log\left(Q_{j}^{-1}\bar{P}\right)+\varepsilon^{2}H\left(Q\right){\bar{P}}^{-1}=0\\ \Rightarrow & \left(1-\varepsilon \right)\varepsilon H\left(Q\right){\bar{P}}^{-1}+\varepsilon \frac{1}{n}\sum_{j=1}^{n}\log\left(Q_{j}^{-1}\bar{P}\right)+\varepsilon^{2}H\left(Q\right){\bar{P}}^{-1}=0 \end{array}$$

Ignore the terms contain $\varepsilon^{2}$ for the constant ε≪1, and we can obtain,

(31) $$\begin{array}{cc} & \varepsilon H\left(Q\right){\bar{P}}^{-1}+\varepsilon \frac{1}{n}\sum_{j=1}^{n}\log\left(Q_{j}^{-1}\bar{P}\right)=0\\ \Rightarrow & H\left(Q\right)=-\frac{1}{n}\sum_{j=1}^{n}\log\left(Q_{j}^{-1}\bar{P}\right)\bar{P} \end{array}$$

☐

**Proposition** **3.** *The influence function of Log-Euclidean mean related to the Log-Euclidean distance, of m HPD matrices $\left\{P_{1},\ldots ,P_{m}\right\}$ and n outliers $\left\{Q\ldots \ldots ,Q_{n}\right\}$ is given by,*

(32) $$\begin{array}{c} H\left(Q\right)=\frac{1}{n}\sum_{j=1}^{n}\left(\log\left(Q_{j}\right)-\log\left(\bar{P}\right)\right)\bar{P} \end{array}$$

**Proof** **of** **Proposition** **3.** Let F(P) be the objection function,

(33) $$\begin{array}{c} F\left(P\right)=\left(1-\varepsilon \right)\frac{1}{m}\sum_{i=1}^{m}∥\log\left(P\right)-\log\left(P_{i}\right){∥}_{F}^{2}+\varepsilon \frac{1}{n}\sum_{j=1}^{n}∥\log\left(P\right)-\log\left(Q_{j}\right){∥}_{F}^{2} \end{array}$$

Note that $\tilde{P}$ is the mean of *m* matrices and *n* outliers, and $\bar{P}$ is the mean of *m* matrices, then, we have,

(34) $$\begin{array}{cc} & \tilde{P}=\underset{P}{\arg\min}\;F\left(P\right)\\ \Rightarrow & \left(1-\varepsilon \right)\frac{1}{m}\sum_{i=1}^{m}2(\log\left(\tilde{P}\right)-\log\left(P_{i}\right){\tilde{P}}^{-1}+\varepsilon \frac{1}{n}\sum_{j=1}^{n}2(\log\left(\tilde{P}\right)-\log\left(Q_{j}\right){\tilde{P}}^{-1}=0\\ \Rightarrow & \left(1-\varepsilon \right)\frac{1}{m}\sum_{i=1}^{m}(\log\left(\tilde{P}\right)-\log\left(P_{i}\right)+\varepsilon \frac{1}{n}\sum_{j=1}^{n}(\log\left(\tilde{P}\right)-\log\left(Q_{j}\right)=0 \end{array}$$

and

(35) $$\begin{array}{cc} & \bar{P}=\underset{P}{\arg\min}\;G\left(P\right),G\left(P\right)=\frac{1}{m}\sum_{i=1}^{m}{∥\log\left(\bar{P}\right)-\log\left(P_{i}\right)∥}_{F}^{2}\\ \Rightarrow & \nabla G\left(P\right)=\frac{1}{m}\sum_{i=1}^{m}2\left(\log\left(\bar{P}\right)-\log\left(P_{i}\right)\right){\bar{P}}^{-1}=0\\ \Rightarrow & \nabla G\left(P\right)=\frac{1}{m}\sum_{i=1}^{m}\left(\log\left(\bar{P}\right)-\log\left(P_{i}\right)\right)=0 \end{array}$$

Using the Taylor expansion on $\tilde{P}=\bar{P}+\varepsilon H\left(Q\right)$, and we have

(36) $$\begin{array}{c} \log\left(\tilde{P}\right)=\log\left(\bar{P}\right)+\varepsilon H\left(Q\right){\bar{P}}^{-1} \end{array}$$

Substitute Equations (35) and (36) into Equation (34), and ignore the terms contain $\varepsilon^{2}$,

(37) $$\begin{array}{cc} & \left(1-\varepsilon \right)\frac{1}{m}\sum_{i=1}^{m}(\log\left(\bar{P}\right)+\varepsilon H\left(Q\right){\bar{P}}^{-1}-\log\left(P_{i}\right)+\varepsilon \frac{1}{n}\sum_{j=1}^{n}(\log\left(\bar{P}\right)+\varepsilon H\left(Q\right){\bar{P}}^{-1}-\log\left(Q_{j}\right)=0\\ \Rightarrow & \left(1-\varepsilon \right)\frac{1}{m}\sum_{i=1}^{m}(\log\left(\bar{P}\right)-\log\left(P_{i}\right)+\left(1-\varepsilon \right)\varepsilon H\left(Q\right){\bar{P}}^{-1}+\varepsilon \frac{1}{n}\sum_{j=1}^{n}(\log\left(\bar{P}\right)-\log\left(Q_{j}\right)+\varepsilon^{2}H\left(Q\right){\bar{P}}^{-1}=0\\ \Rightarrow & \varepsilon H\left(Q\right){\bar{P}}^{-1}+\varepsilon \frac{1}{n}\sum_{j=1}^{n}(\log\left(\bar{P}\right)-\log\left(Q_{j}\right)=0\\ \Rightarrow & H\left(Q\right)=\frac{1}{n}\sum_{j=1}^{n}(\log\left(Q_{j}-\log\left(\bar{P}\right)\right)\bar{P} \end{array}$$

☐

**Proposition** **4.** *The influence function of Hellinger mean related to the Hellinger distance, of m HPD matrices $\left\{P_{1},\ldots ,P_{m}\right\}$ and n outliers $\left\{Q\ldots \ldots ,Q_{n}\right\}$ is given by,*

(38) $$\begin{array}{cc} H(Q)= & \left\{\frac{1}{n}\sum_{j=1}^{n}{|Q_{j}|}^{1/4\;}{|\frac{\bar{P}+Q_{j}}{2}|}^{-1/2\;}\left({\left(\frac{\bar{P}+Q_{j}}{2}\right)}^{-1}-{\bar{P}}^{-1}\right)\right\}\\ \times & {\left\{\frac{1}{m}\sum_{i=1}^{n}{|P_{i}|}^{1/4\;}{|\frac{\bar{P}+P_{i}}{2}|}^{-1/2\;}{\left({\bar{P}}^{-1}-\frac{1}{2}{\left(\frac{\bar{P}+P_{i}}{2}\right)}^{-1}\right)}^{2}\right\}}^{-1} \end{array}$$

**Proof** **of** **Proposition** **4.** Let F(P) be the objection function,

(39) $$\begin{array}{c} F\left(P\right)=\left(1-\varepsilon \right)\frac{1}{m}\sum_{i=1}^{m}\left(1-{\left|P\right|}^{1/4\;}{|P_{i}|}^{1/4\;}{|\frac{P+P_{i}}{2}|}^{-1/2\;}\right)+\varepsilon \frac{1}{n}\sum_{j=1}^{n}\left(1-{\left|P\right|}^{1/4\;}{|Q_{j}|}^{1/4\;}{|\frac{P+Q_{j}}{2}|}^{-1/2\;}\right) \end{array}$$

Note that $\tilde{P}$ is the mean of *m* matrices and *n* outliers, and $\bar{P}$ is the mean of *m* matrices, then, we have,

(40) $$\begin{array}{cc} & \tilde{P}=\underset{P}{\arg\min}\;F\left(P\right),\nabla F\left(\tilde{P}\right)=0\\ \Rightarrow - & \left(1-\varepsilon \right)\frac{1}{m}\sum_{i=1}^{m}\left(\frac{1}{4}{\left|\tilde{P}\right|}^{1/4\;}{\left|P_{i}\right|}^{1/4\;}{\left|\frac{\tilde{P}+P_{i}}{2}\right|}^{-1/2\;}{\tilde{P}}^{-1}-\frac{1}{4}{\left|\tilde{P}\right|}^{1/4\;}{\left|P_{i}\right|}^{1/4\;}{\left|\frac{\tilde{P}+P_{i}}{2}\right|}^{-1/2\;}{\left(\frac{\tilde{P}+P_{i}}{2}\right)}^{-1}\right)\\ - & \varepsilon \frac{1}{n}\sum_{j=1}^{n}\left(\frac{1}{4}{\left|\tilde{P}\right|}^{1/4\;}{\left|Q_{j}\right|}^{1/4\;}{\left|\frac{\tilde{P}+Q_{j}}{2}\right|}^{-1/2\;}{\tilde{P}}^{-1}-\frac{1}{4}{\left|\tilde{P}\right|}^{1/4\;}{\left|Q_{j}\right|}^{1/4\;}{\left|\frac{\tilde{P}+Q_{j}}{2}\right|}^{-1/2\;}{\left(\frac{\tilde{P}+Q_{j}}{2}\right)}^{-1}\right)=0\\ \Rightarrow & \left(1-\varepsilon \right)\frac{1}{m}\sum_{i=1}^{m}\left({\left|P_{i}\right|}^{1/4\;}{\left|\frac{\tilde{P}+P_{i}}{2}\right|}^{-1/2\;}{\tilde{P}}^{-1}-{\left|P_{i}\right|}^{1/4\;}{\left|\frac{\tilde{P}+P_{i}}{2}\right|}^{-1/2\;}{\left(\frac{\tilde{P}+P_{i}}{2}\right)}^{-1}\right)\\ + & \varepsilon \frac{1}{n}\sum_{j=1}^{n}\left({\left|Q_{j}\right|}^{1/4\;}{\left|\frac{\tilde{P}+Q_{j}}{2}\right|}^{-1/2\;}{\tilde{P}}^{-1}-{\left|Q_{j}\right|}^{1/4\;}{\left|\frac{\tilde{P}+Q_{j}}{2}\right|}^{-1/2\;}{\left(\frac{\tilde{P}+Q_{j}}{2}\right)}^{-1}\right)=0 \end{array}$$

and

(41) $$\begin{array}{cc} & \bar{P}=\underset{P}{\arg\min}\;G\left(P\right),G\left(P\right)=\frac{1}{m}\sum_{i=1}^{m}\left(1-{\left|P\right|}^{1/4\;}{\left|P_{i}\right|}^{1/4\;}{\left|\frac{P+P_{i}}{2}\right|}^{-1/2\;}\right)\\ \Rightarrow & \nabla G\left(\bar{P}\right)=\frac{1}{m}\sum_{i=1}^{m}\left(\frac{1}{4}{\left|\bar{P}\right|}^{1/4\;}{\left|P_{i}\right|}^{1/4\;}{\left|\frac{\bar{P}+P_{i}}{2}\right|}^{-1/2\;}{\bar{P}}^{-1}-\frac{1}{4}{\left|\bar{P}\right|}^{1/4\;}{\left|P_{i}\right|}^{1/4\;}{\left|\frac{\bar{P}+P_{i}}{2}\right|}^{-1/2\;}{\left(\frac{\bar{P}+P_{i}}{2}\right)}^{-1}\right)=0\\ \Rightarrow & \frac{1}{m}\sum_{i=1}^{m}\left({\left|P_{i}\right|}^{1/4\;}{\left|\frac{\bar{P}+P_{i}}{2}\right|}^{-1/2\;}{\bar{P}}^{-1}-{\left|P_{i}\right|}^{1/4\;}{\left|\frac{\bar{P}+P_{i}}{2}\right|}^{-1/2\;}{\left(\frac{\bar{P}+P_{i}}{2}\right)}^{-1}\right)=0 \end{array}$$

Using the Taylor expansion on $\tilde{P}=\bar{P}+\varepsilon H\left(Q\right)$, and we have

(42) $$\begin{array}{c} {\left|\frac{\tilde{P}+P_{i}}{2}\right|}^{-1/2\;}={\left|\frac{\bar{P}+P_{i}}{2}\right|}^{-1/2\;}-\frac{1}{4}\varepsilon H\left(Q\right){\left|\frac{\bar{P}+P_{i}}{2}\right|}^{-1/2\;}{\left(\frac{\bar{P}+P_{i}}{2}\right)}^{-1}\\ {\tilde{P}}^{-1}={\bar{P}}^{-1}-\varepsilon H\left(Q\right){\bar{P}}^{-2}\\ {\left(\frac{\tilde{P}+P_{i}}{2}\right)}^{-1}={\left(\frac{\bar{P}+P_{i}}{2}\right)}^{-1}-\frac{1}{2}\varepsilon H\left(Q\right){\left(\frac{\bar{P}+P_{i}}{2}\right)}^{-2} \end{array}$$

Substitute Equations (41) and (42) into Equation (40), and ignore the terms contain $\varepsilon^{2}$,

(43) $$\begin{array}{cc} & \left(1-\varepsilon \right)\frac{1}{m}\sum_{i=1}^{m}\left(\right|P_{i}{|}^{1/4}\left(\right|\frac{1}{2}\left(\bar{P}+P_{i}\right){|}^{-1/2}-\frac{1}{4}\varepsilon H\left(Q\right)|\frac{1}{2}\left(\bar{P}+P_{i}\right){|}^{-1/2}{\left(\frac{1}{2}\left(\bar{P}+P_{i}\right)\right)}^{-1})\\ \times & \left({\bar{P}}^{-1}-\varepsilon H\left(Q\right){\bar{P}}^{-2}-{\left(\frac{1}{2}\left(\bar{P}+P_{i}\right)\right)}^{-1}+\frac{1}{2}\varepsilon H\left(Q\right){\left(\frac{1}{2}\left(\bar{P}+P_{i}\right)\right)}^{-2})\right)\\ + & \varepsilon \frac{1}{n}\sum_{j=1}^{n}\left(\right|Q_{j}{|}^{1/4}\left(\right|\frac{1}{2}\left(\bar{P}+Q_{j}\right){|}^{-1/2}-\frac{1}{4}\varepsilon H\left(Q\right)|\frac{1}{2}\left(\bar{P}+Q_{j}\right){|}^{-1/2}{\left(\frac{1}{2}\left(\bar{P}+Q_{j}\right)\right)}^{-1})\\ \times & ({\bar{P}}^{-1}-\varepsilon H\left(Q\right){\bar{P}}^{-2}-{\left(\frac{1}{2}\left(\bar{P}+Q_{j}\right)\right)}^{-1}+\frac{1}{2}\varepsilon H\left(Q\right){\left(\frac{1}{2}\left(\bar{P}+Q_{j}\right)\right)}^{-2}))=0\\ \Rightarrow & \varepsilon H\left(Q\right)\frac{1}{m}\sum_{i=1}^{m}{\left|P_{i}\right|}^{1/4\;}{\left|\frac{\bar{P}+P_{i}}{2}\right|}^{-1/2\;}{\left({\bar{P}}^{-1}-\frac{1}{2}{\left(\frac{\bar{P}+P_{i}}{2}\right)}^{-1}\right)}^{2}\\ = & \varepsilon \frac{1}{n}\sum_{j=1}^{n}{\left|Q_{j}\right|}^{1/4\;}{\left|\frac{\bar{P}+Q_{j}}{2}\right|}^{-1/2\;}\left({\left(\frac{\bar{P}+Q_{j}}{2}\right)}^{-1}-{\bar{P}}^{-1}\right)\\ \Rightarrow H(Q)= & \left\{\frac{1}{n}\sum_{j=1}^{n}{|Q_{j}|}^{1/4\;}{|\frac{\bar{P}+Q_{j}}{2}|}^{-1/2\;}\left({\left(\frac{\bar{P}+Q_{j}}{2}\right)}^{-1}-{\bar{P}}^{-1}\right)\right\}\\ \times & {\left\{\frac{1}{m}\sum_{i=1}^{n}{|P_{i}|}^{1/4\;}{|\frac{\bar{P}+P_{i}}{2}|}^{-1/2\;}{\left({\bar{P}}^{-1}-\frac{1}{2}{\left(\frac{\bar{P}+P_{i}}{2}\right)}^{-1}\right)}^{2}\right\}}^{-1} \end{array}$$

☐

**Proposition** **5.** *The influence function of KL mean related to the KL divergence, of m HPD matrices $\left\{P_{1},\ldots ,P_{m}\right\}$ and n outliers $\left\{Q\ldots \ldots ,Q_{n}\right\}$ is given by,*

(44) $$\begin{array}{c} H\left(Q\right)=\bar{P}-\frac{1}{n}\sum_{j=1}^{n}Q_{j}^{-1}{\bar{P}}^{2} \end{array}$$

**Proof** **of** **Proposition** **5.** Let F(P) be the objection function,

(45) $$\begin{array}{c} F\left(P\right)=\left(1-\varepsilon \right)\frac{1}{m}\sum_{i=1}^{m}tr(P_{i}^{-1}P-\log\left(P_{i}^{-1}P\right)-I)+\varepsilon \frac{1}{n}\sum_{j=1}^{n}tr(Q_{j}^{-1}P-\log\left(Q_{j}^{-1}P\right)-I) \end{array}$$

As $\tilde{P}$ is the mean of *m* matrices and *n* outliers, and $\bar{P}$ is the mean of *m* matrices, then, we have,

(46) $$\begin{array}{cc} & \tilde{P}=\underset{P}{\arg\min}\;F\left(P\right),\nabla F\left(\tilde{P}\right)=0\\ \Rightarrow & \left(1-\varepsilon \right)\frac{1}{m}\sum_{i=1}^{m}\left(P_{i}^{-1}-{\tilde{P}}^{-1}\right)+\varepsilon \frac{1}{n}\sum_{j=1}^{n}\left(Q_{j}^{-1}-{\tilde{P}}^{-1}\right)=0 \end{array}$$

and

(47) $$\begin{array}{cc} & \bar{P}=\underset{P}{\arg\min}\;G\left(P\right),G\left(P\right)=\frac{1}{m}\sum_{i=1}^{m}tr(P_{i}^{-1}P-\log\left(P_{i}^{-1}P\right)-I)\\ \Rightarrow & \nabla G\left(\bar{P}\right)=0\Rightarrow \frac{1}{m}\sum_{i=1}^{m}\left(P_{i}^{-1}-{\bar{P}}^{-1}\right)=0 \end{array}$$

Using the Taylor expansion on $\tilde{P}=\bar{P}+\varepsilon H\left(Q\right)$, and we have,

(48) $$\begin{array}{c} {\tilde{P}}^{-1}={\bar{P}}^{-1}-\varepsilon H\left(Q\right){\bar{P}}^{-2} \end{array}$$

Substitute Equations (47) and (48) into Equation (46), and ignore the terms contain $\varepsilon^{2}$,

(49) $$\begin{array}{cc} & \left(1-\varepsilon \right)\frac{1}{m}\sum_{i=1}^{m}\left(P_{i}^{-1}-{\bar{P}}^{-1}+\varepsilon H\left(Q\right){\bar{P}}^{-2}\right)+\varepsilon \frac{1}{n}\sum_{j=1}^{n}\left(Q_{j}^{-1}-{\bar{P}}^{-1}+\varepsilon H\left(Q\right){\bar{P}}^{-2}\right)=0\\ \Rightarrow & \left(1-\varepsilon \right)\varepsilon H\left(Q\right){\bar{P}}^{-2}+\varepsilon \frac{1}{n}\sum_{j=1}^{n}\left(Q_{j}^{-1}-{\bar{P}}^{-1}\right)=0\\ \Rightarrow & H\left(Q\right){\bar{P}}^{-2}=\frac{1}{n}\sum_{j=1}^{n}\left({\bar{P}}^{-1}-Q_{j}^{-1}\right)\\ \Rightarrow & H\left(Q\right)=\bar{P}-\frac{1}{n}\sum_{j=1}^{n}Q_{j}^{-1}{\bar{P}}^{2} \end{array}$$

☐

**Proposition** **6.** *The influence function of Bhattacharyya mean related to the Bhattacharyya divergence, of m HPD matrices $\left\{P_{1},\ldots ,P_{m}\right\}$ and n outliers $\left\{Q\ldots \ldots ,Q_{n}\right\}$ is given by,*

(50) $$\begin{array}{c} H\left(Q\right)=\left\{\frac{1}{n}\sum_{j=1}^{n}\left({\bar{P}}^{-1}-{\left(\frac{\bar{P}+Q_{j}}{2}\right)}^{-1}\right)\right\}{\left\{\frac{1}{m}\sum_{i=1}^{m}\left({\bar{P}}^{-2}-\frac{1}{2}{\left(\frac{\bar{P}+P_{i}}{2}\right)}^{-2}\right)\right\}}^{-1} \end{array}$$

**Proof** **of** **Proposition** **6.** Let F(P) be the objection function,

(51) $$F\left(P\right)=\left(1-\varepsilon \right)\frac{1}{m}\sum_{i=1}^{m}4\left(\log\left|\frac{P+P_{i}}{2}\right|-\frac{1}{2}\log\left|P\right|\left|P_{i}\right|\right)+\varepsilon \frac{1}{n}\sum_{j=1}^{n}4\left(\log\left|\frac{P+Q_{j}}{2}\right|-\frac{1}{2}\log\left|P\right|\left|Q_{j}\right|\right)$$

Note that $\tilde{P}$ is the mean of *m* matrices and *n* outliers, and $\bar{P}$ is the mean of *m* matrices, then, we have,

(52) $$\begin{array}{cc} & \tilde{P}=\underset{P}{\arg\min}\;F\left(P\right),\nabla F\left(\tilde{P}\right)=0\\ \Rightarrow & \left(1-\varepsilon \right)\frac{1}{m}\sum_{i=1}^{m}4\left({\left(\frac{\tilde{P}+P_{i}}{2}\right)}^{-1}-{\tilde{P}}^{-1}\right)+\varepsilon \frac{1}{n}\sum_{j=1}^{n}4\left({\left(\frac{\tilde{P}+Q_{j}}{2}\right)}^{-1}-{\tilde{P}}^{-1}\right)=0 \end{array}$$

and

(53) $$\begin{array}{cc} & \bar{P}=\underset{P}{\arg\min}\;G\left(P\right),G\left(P\right)=\frac{1}{m}\sum_{i=1}^{m}4(\log|\frac{P+P_{i}}{2}|-\frac{1}{2}\log|P||P_{i}|)\\ \Rightarrow & \frac{1}{m}\sum_{i=1}^{m}4({\left(\frac{\bar{P}+P_{i}}{2}\right)}^{-1}-{\bar{P}}^{-1})=0 \end{array}$$

Using the Taylor expansion on $\tilde{P}=\bar{P}+\varepsilon H\left(Q\right)$, and we have,

(54) $$\begin{array}{c} {\left(\frac{\tilde{P}+P_{i}}{2}\right)}^{-1}={\left(\frac{\bar{P}+P_{i}}{2}\right)}^{-1}-\frac{1}{2}\varepsilon H\left(Q\right){\left(\frac{\bar{P}+P_{i}}{2}\right)}^{-2}\\ {\left(\frac{\tilde{P}+Q_{j}}{2}\right)}^{-1}={\left(\frac{\bar{P}+Q_{j}}{2}\right)}^{-1}-\frac{1}{2}\varepsilon H\left(Q\right){\left(\frac{\bar{P}+Q_{j}}{2}\right)}^{-2}\\ {\tilde{P}}^{-1}={\bar{P}}^{-1}-\varepsilon H\left(Q\right){\bar{P}}^{-2} \end{array}$$

Substitute Equations (53) and (54) into Equation (52), and ignore the terms contain $\varepsilon^{2}$,

(55) $$\begin{array}{cc} & \left(1-\varepsilon \right)\frac{1}{m}\sum_{i=1}^{m}\left({\left(\frac{\bar{P}+P_{i}}{2}\right)}^{-1}-\frac{1}{2}Hz\left(Q\right){\left(\frac{\bar{P}+P_{i}}{2}\right)}^{-2}-{\bar{P}}^{-1}+Hz\left(Q\right){\bar{P}}^{-2}\right)\\ + & \varepsilon \frac{1}{n}\sum_{j=1}^{n}\left({\left(\frac{\bar{P}+Q_{j}}{2}\right)}^{-1}-\frac{1}{2}Hz\left(Q\right){\left(\frac{\bar{P}+Q_{j}}{2}\right)}^{-2}-{\bar{P}}^{-1}+Hz\left(Q\right){\bar{P}}^{-2}\right)=0\\ \Rightarrow & \frac{1}{m}\sum_{i=1}^{m}\left(\varepsilon H\left(Q\right){\bar{P}}^{-2}-\frac{1}{2}\varepsilon H\left(Q\right){\left(\frac{\bar{P}+P_{i}}{2}\right)}^{-2}\right)+\varepsilon \frac{1}{n}\sum_{j=1}^{n}\left({\left(\frac{\bar{P}+Q_{j}}{2}\right)}^{-1}-{\bar{P}}^{-1}\right)=0\\ \Rightarrow & H\left(Q\right)\frac{1}{m}\sum_{i=1}^{m}\left({\bar{P}}^{-2}-\frac{1}{2}{\left(\frac{\bar{P}+P_{i}}{2}\right)}^{-2}\right)=\frac{1}{n}\sum_{j=1}^{n}\left({\bar{P}}^{-1}-{\left(\frac{\bar{P}+Q_{j}}{2}\right)}^{-1}\right)\\ \Rightarrow & H\left(Q\right)=\left\{\frac{1}{n}\sum_{j=1}^{n}\left({\bar{P}}^{-1}-{\left(\frac{\bar{P}+Q_{j}}{2}\right)}^{-1}\right)\right\}{\left\{\frac{1}{m}\sum_{i=1}^{m}\left({\bar{P}}^{-2}-\frac{1}{2}{\left(\frac{\bar{P}+P_{i}}{2}\right)}^{-2}\right)\right\}}^{-1} \end{array}$$

☐

**Proposition** **7.** *The influence function of SKL mean related to the SKL divergence, of m HPD matrices $\left\{P_{1},\ldots ,P_{m}\right\}$ and n outliers $\left\{Q\ldots \ldots ,Q_{n}\right\}$ is given by,*

(56) $$\begin{array}{c} H\left(Q\right)={\left(\frac{2}{m}\sum_{i=1}^{m}P_{i}\right)}^{-1}\left(\frac{1}{n}\sum_{j=1}^{n}\left(\bar{P}Q_{j}-Q_{j}^{-1}{\bar{P}}^{3}\right)\right) \end{array}$$

**Proof** **of** **Proposition** **7.** Let F(P) be the objection function,

(57) $$\begin{array}{c} F\left(P\right)=\left(1-\varepsilon \right)\frac{1}{m}\sum_{i=1}^{m}tr\left(P_{i}^{-1}P+P^{-1}P_{i}-2I\right)+\varepsilon \frac{1}{n}\sum_{j=1}^{n}tr\left(Q_{j}^{-1}P+P^{-1}Q_{j}-2I\right) \end{array}$$

Note that $\tilde{P}$ is the mean of *m* matrices and *n* outliers, and $\bar{P}$ is the mean of *m* matrices, then, we have,

(58) $$\begin{array}{cc} & \tilde{P}=\underset{P}{\arg\min}\;F\left(P\right),\nabla F\left(\tilde{P}\right)=0\\ \Rightarrow & \left(1-\varepsilon \right)\frac{1}{m}\sum_{i=1}^{m}\left(P_{i}^{-1}-{\tilde{P}}^{-2}P_{i}\right)+\varepsilon \frac{1}{n}\sum_{j=1}^{n}\left(Q_{j}^{-1}-{\tilde{P}}^{-2}Q_{j}\right)=0 \end{array}$$

and

(59) $$\begin{array}{cc} & \bar{P}=\underset{P}{\arg\min}\;G\left(P\right),G\left(P\right)=\frac{1}{m}\sum_{i=1}^{m}tr\left(P_{i}^{-1}P+P^{-1}P_{i}-2I\right)\\ \Rightarrow & \frac{1}{m}\sum_{i=1}^{m}\left(P_{i}^{-1}-{\bar{P}}^{-2}P_{i}\right)=0 \end{array}$$

Using the Taylor expansion on $\tilde{P}=\bar{P}+\varepsilon H\left(Q\right)$, and we have,

(60) $$\begin{array}{c} {\tilde{P}}^{-2}={\bar{P}}^{-2}-2\varepsilon H\left(Q\right){\bar{P}}^{-3} \end{array}$$

Substitute Equations (59) and (60) into Equation (58), and ignore the terms contain $\varepsilon^{2}$,

(61) $$\begin{array}{cc} & \left(1-\varepsilon \right)\frac{1}{m}\sum_{i=1}^{m}\left(P_{i}^{-1}-\left({\bar{P}}^{-2}-2\varepsilon H\left(Q\right){\bar{P}}^{-3}\right)P_{i}\right)+\varepsilon \frac{1}{n}\sum_{j=1}^{n}\left(Q_{j}^{-1}-\left({\bar{P}}^{-2}-2\varepsilon H\left(Q\right){\bar{P}}^{-3}\right)Q_{j}\right)=0\\ \Rightarrow & \frac{1}{m}\sum_{i=1}^{m}\left(2\varepsilon H\left(Q\right){\bar{P}}^{-3}P_{i}\right)+\varepsilon \frac{1}{n}\sum_{j=1}^{n}\left(Q_{j}^{-1}-{\bar{P}}^{-2}Q_{j}\right)=0\\ \Rightarrow & H\left(Q\right)={\left(\frac{2}{m}\sum_{i=1}^{m}P_{i}\right)}^{-1}\left(\frac{1}{n}\sum_{j=1}^{n}\left(\bar{P}Q_{j}-Q_{j}^{-1}{\bar{P}}^{3}\right)\right) \end{array}$$

☐

## 5. Numerical Simulations

In order to gain a better understanding of the superiority of proposed estimators, simulation results of the performance of an ANMF with the proposed estimator in heterogeneous clutter are presented. As there is not an analytical expression for the detection threshold, the standard Monte Carlo technique [34] is utilized. A similar approach was recently used to solve several problems from different areas, such as physics [35], decision theory [36], engineering [37], computational geometry [38], finance [39], etc. The rule of adaptive normalized matched filter (ANMF) is given as [40],

(62) $$\begin{array}{c} \frac{s^{H}{\hat{\Sigma }}^{-1}r}{\left(s^{H}{\hat{\Sigma }}^{-1}s\right)\left(r^{H}{\hat{\Sigma }}^{-1}r\right)}\underset{H_{0}}{\overset{H_{1}}{≷}}\gamma \end{array}$$

where $\hat{\Sigma }$ is the clutter covariance estimation. r is the sample data in the cell under test. γ denotes the threshold, which is derived by Monte Carlo method in order to maintain the false alarm constant. s is the target steering vector, and is given by,

(63) $$\begin{array}{c} s=\frac{1}{\sqrt{N}}{\left[1,\exp\left(j2\pi f_{d}\right),\ldots ,\exp\left(j2\pi \left(N-1\right)f_{d}\right)\right]}^{T} \end{array}$$

where $f_{d}$ is the normalized Doppler frequency. According to Equation (2), the terms $r_{k},\;k\;=\;1,\ldots ,\;K$ are compound-Gaussian random vectors, and sharing the same covariance matrix Σ,

(64) $$\begin{array}{c} \Sigma \;=\;\Sigma_{0}\;+\;I \end{array}$$

where I is accounting for the thermal noise. $\Sigma_{0}$ is related to the clutter, modeled as,

(65) $$\begin{array}{c} \Sigma_{0}\left(i,k\right)=\sigma_{c}^{2}\rho^{\left|i--k\right|}e^{j2\pi f_{dc}\left(i--k\right)},\;i,k\;=\;1,\ldots N \end{array}$$

where ρ is the one-lag correlation coefficient. $\sigma_{c}$ is the clutter-to-noise power ratio. $f_{dc}$ is the clutter normalized Doppler frequency.

In addition, τ and $\tau_{k}$ are positive and real independent and identical distributed random variables, and are assumed to follow the inverse gamma distribution,

(66) $$\begin{array}{c} f\left(x\right)=\frac{\beta^{\alpha }}{\Gamma \left(\alpha \right)}x^{-\alpha -1}\exp\left(-\frac{\beta }{x}\right),x\geq 0 \end{array}$$

where α and β denote the shape and scale parameters, respectively. $\Gamma \left(\cdot \right)$ is the gamma function. In the simulation, we set ρ=0.9, $f_{dc}\;=\;0.1$, and $\sigma_{c}^{2}\;=\;25$ dB. The parameters α=3, and β=1.

**Figure 2:**
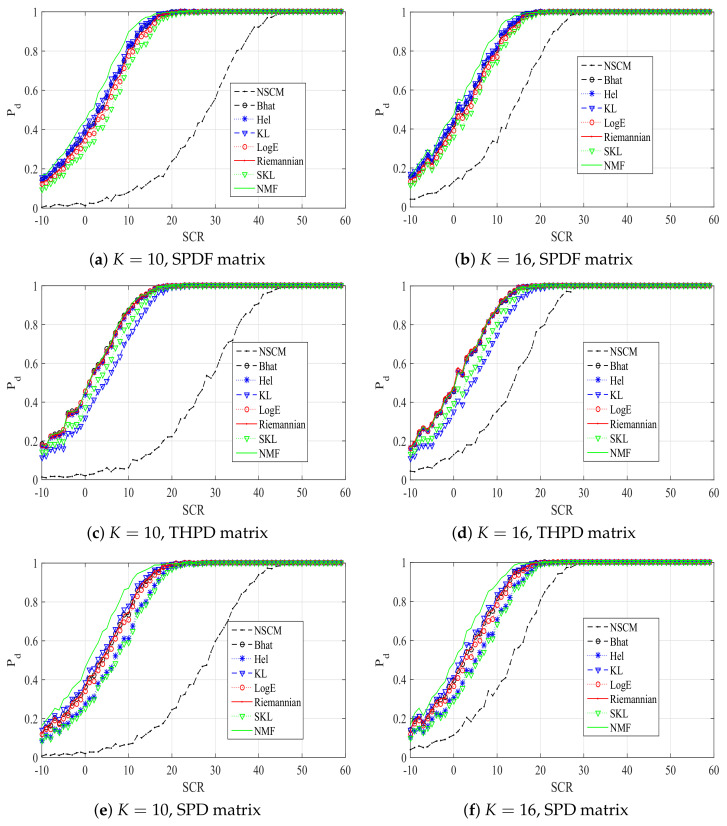
$P_{d}$ versus SCR plots of ANMFs with proposed estimators, the NSCM estimator, and NMF.

In the following, we analyze the performance of an ANMF with the proposed estimators, in terms of detection probability ($P_{d}$), also in comparison with the optimum detector, which assumes the perfect knowledge of the disturbance covariance matrix, NMF. In particular, the positive-definite matrix obtained using (1), (2), and (3) is noted as the SPDF, THPD, and SPD matrix, respectively. Simulation results are shown in Figure 2 with N=8, $P_{fa}=10^{-4}$, it is clear that the proposed estimators have different performances, and all the proposed estimators have better performances than the NSCM estimator when K=10 and K=16. In particular, our proposed estimators have different performance when different positive-definite matrix is utilized. For the SPDF positive-definite matrix, the Bhat estimator, the Hel estimator, and the KL estimator have comparable performances, and outperform others. The SKL estimator has the worst performance, while the KL estimator has the best performance. However, this relationship is different on the condition of the THPD positive-definite matrix. Particularly, performances of the KL estimator and the SKL estimator are poor. Performances of the other proposed estimators are close to the optimum. For the SPD positive-definite matrix, relationships of proposed estimators are similar to the case of SPDF positive-definite matrix. The KL estimator has the best performance, while the performance of SKL estimator is the worst. In addition, the performance of Hel estimator is poor. These results imply that performances of proposed estimators are related to the used positive-definite matrix.

**Figure 3:**
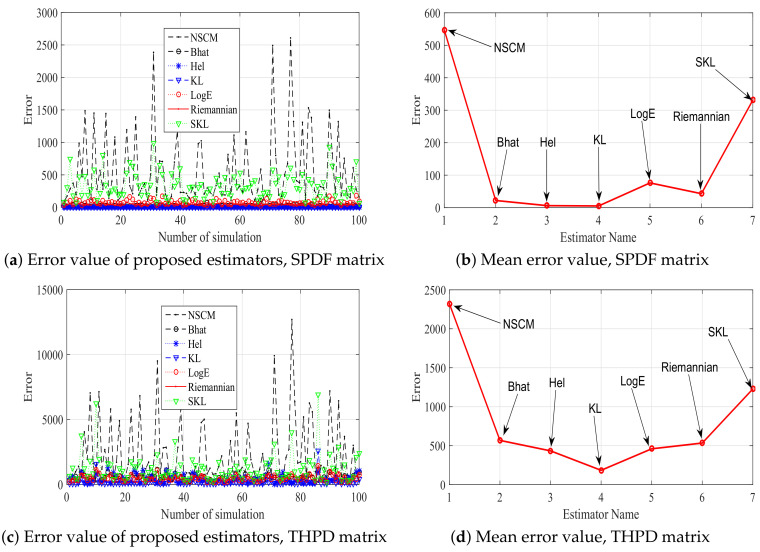

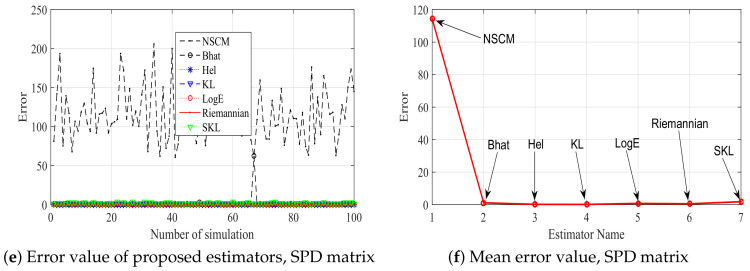
The error value of proposed estimators and their corresponding mean vlaue.

In order to show the influence function of robustness of proposed estimators, 17 positive-definite matrices with an injected outlier are considered. The value of influence function is computed as Propositions 1–7, and 100 times simulations are repeated. A total of 100 simulation results and the average of values of influence function are shown in Figure 3. From Figure 3 we can know that our proposed estimators are more robust than the NSCM estimator. In particular, the robustness of SKL estimator is poor, while the KL estimator has the best robustness when the SPDF or THPD positive-definite matrix is utilized. For the SPD positive-definite matrix, all proposed estimators have comparable robustness. It can be concluded that the robustness of proposed estimators is related to the used positive-definite matrix. It is worth pointing out that the three HPD matrices, namely the SPDF, THPD, and SPD matrix, have different structures. Both the SPDF and the SPD matrices have a largest eigenvalue and N−1 equal eigenvalues. Their differences lie in the multiple between the maximum eigenvalue and the minimum eigenvalue. In particular, this multiple of the SPD matrix is the constant number $\kappa_{M}$, while the SPDF matrix has a varied multiple. For the THPD matrix, all eigenvalues are different. Riemannian manifolds composed of different positive-definite matrices have different geometric structures. Thus, the estimators associated with different metrics on Riemannian manifold may have different behaviors.

**Figure 4:**
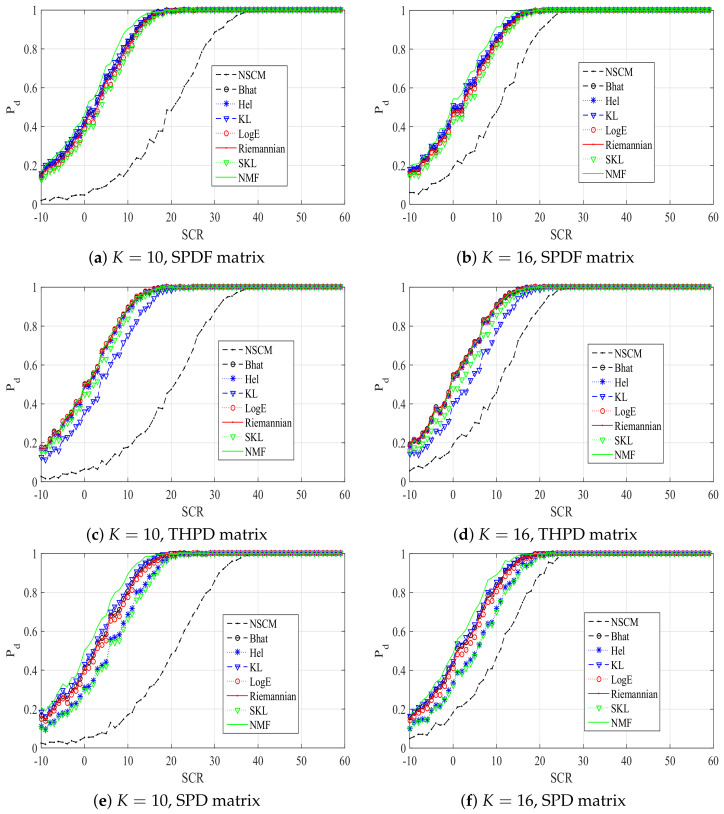
$P_{d}$ versus SCR plots of ANMFs with proposed estimators, the NSCM estimator, and NMF in a contaminated environment.

Figure 4 plots the $P_{d}$ of the ANMF with our proposed estimators, the NSCM estimator, and the NMF detector in a contaminated clutter. An outlier is injected in one reference cell, the number of reference cell *K* is set to 10 and 16, respectively. The dimension of the sample data is 8. It can be noted from Figure 4 that performances of our proposed estimators have not been significantly reduced, while there is a degradation in the performance of the NSCM estimator. Relationships of performances are similar to curves of Figure 2. These results prove the advantage of our proposed estimators sufficiently.

## 6. Conclusions

In this paper, a class of covariance estimators based on information divergences is proposed in heterogeneous clutter. Particularly, the problem of disturbance covariance estimation is reformulated as obtaining the geometric mean on the Riemannian manifold. Six mean estimators related to information measures are derived. Moreover, the robustness of proposed estimators are analyzed via the influence function, and the analytic expression of influence function is deduced. At the analysis stage, the performance advantage and robustness of our proposed estimators are verified by means of simulation results in heterogeneous environment.

## Figures and Tables

**Table 1:** Geometric means related to different measures.

Geometric Measure	Mean
Riemannian	${\bar{P}}_{t+1}={\bar{P}}_{t}^{{}^{1/2\;}}\exp\left\{\varepsilon_{t}\left(\sum_{k=1}^{K}\log\left({\bar{P}}_{t}^{{}^{-1/2\;}}P_{k}{\bar{P}}_{t}^{{}^{-1/2\;}}\right)\right)\right\}{\bar{P}}_{t}^{{}^{1/2\;}}$
Log-Euclidean	$\bar{P}=\exp\left(\frac{1}{K}\sum_{i=1}^{K}\log(P_{i})\right)$
Hellinger	${\bar{P}}_{t+1}={\left(\frac{\sum_{i=1}^{K}{\left|{\bar{P}}_{t}\right|}^{1/4\;}{\left|P_{i}\right|}^{1/4\;}{\left|\frac{1}{2}\left({\bar{P}}_{t}+P_{i}\right)\right|}^{-1/2\;}{\left(\frac{1}{2}\left({\bar{P}}_{t}+P_{i}\right)\right)}^{-1}}{\left(\sum_{i=1}^{K}{\left|{\bar{P}}_{t}\right|}^{1/4\;}{\left|P_{i}\right|}^{1/4\;}{\left|\frac{1}{2}\left({\bar{P}}_{t}+P_{i}\right)\right|}^{-1/2\;}\right)}\right)}^{-1}$
KL	$\bar{P}={\left(\frac{1}{K}\sum_{i=1}^{K}P_{i}^{-1}\right)}^{-1}$
Bhattacharyya	${\bar{P}}_{t+1}={\left(\frac{1}{K}\sum_{i=1}^{K}{\left(\frac{{\bar{P}}_{t}+P_{i}}{2}\right)}^{-1}\right)}^{-1}$
SKL	$\bar{P}={\left(\left(\frac{1}{K}\sum_{i=1}^{K}P_{i}\right){\left(\frac{1}{K}\sum_{k=1}^{K}P_{k}^{-1}\right)}^{-1}\right)}^{1/2\;}$

Where *t* is the number of iteration, and $\varepsilon_{t}$ is is the step size of iteration.
